# Pneumonia in lung transplant recipients: the role of donor, recipient, and hospital-acquired microorganisms

**DOI:** 10.36416/1806-3756/e20250468

**Published:** 2026-05-22

**Authors:** Alexandre Mestre Tejo, Flávio Pola dos Reis, Ana Carolina de Avila, Mauro Razuk, Samuel Lucas dos Santos, Luis Gustavo Abdalla, Lucas Matos Fernandes, Silvia Vidal Campos, Paulo Manuel Pêgo-Fernandes

**Affiliations:** 1. Departamento de Infectologia e Medicina Tropical, Hospital das Clínicas, Faculdade de Medicina, Universidade de São Paulo, São Paulo (SP) Brasil.; 2. Departamento de Cirurgia Torácica, Instituto do Coração, Hospital das Clínicas, Faculdade de Medicina, Universidade de São Paulo, São Paulo (SP) Brasil.; 3. Latner Thoracic Research Laboratories, Toronto General Hospital Research Institute, University Health Network, Toronto (ON) Canada.

**Keywords:** Lung transplantation, Pneumonia, Infectious disease transmission

## Abstract

**Objective:**

Donor-derived disease is an inherent risk in solid organ transplantation, especially lung transplantation (LTx), because of direct contact between the organ and the external environment, and the consequences of lung denervation after LTx. We sought to demonstrate the burden of pneumonia after LTx in a large center in Brazil, characterizing the main source of infection and its associated mortality.

**Methods:**

We performed a retrospective analysis of all LTx procedures occurring between 2017 and 2023 at a public center in Brazil. All microorganisms isolated from the donor BAL fluid and the recipient bronchial secretions at organ harvest were compared with those found by postoperative day 14 and classified as donor-derived, recipient colonization-related, or hospital-acquired pneumonia. We analyzed the characteristics of donors and recipients, as well as the associated mortality.

**Results:**

Donor-derived pneumonia was found in 8/149 patients (5.36%) who received lungs from infected donors. In all cases, donor-derived pneumonia was due to gram-negative bacilli, all of which showed drug resistance, causing a 50% mortality rate. Recipient colonization-related pneumonia occurred in 14/107 patients (13%), being mostly due to Pseudomonas aeruginosa (in 58.8%), with a 28.6% risk of death. Hospital-acquired pneumonia occurred in 17 of 217 recipients (7.83%), being mainly due to Acinetobacter baumannii (in 20%), P. aeruginosa (in 15%), Klebsiella pneumoniae (in 10%), Stenotrophomonas maltophilia (in 10%), or Burkholderia cepacia (in 10%), with a mortality rate of 50%.

**Conclusions:**

Although infrequent, donor-derived pneumonia appears to be associated with high early mortality, comparable to that for hospital-acquired pneumonia. Recipient colonization-related and hospital-acquired pneumonia are linked to an increased chance of death, especially in patients colonized with P. aeruginosa and those with extensively drug-resistant bacteria, respectively.

## INTRODUCTION

Donor-derived infections are an inherent risk of solid organ transplantation. According to the U.S. Organ Procurement and Transplantation Network Disease Transmission Advisory Committee, the rate of infection transmitted in solid organ transplantation is 23.0/10,000, with a related mortality rate of 15%.^1^ Several factors impact the risk of infection after solid organ transplantation, including previous donor exposure, previous recipient exposure, and the net state of immunosuppression, which includes multiple immunosuppressive therapy effects, as well as the prior immune status of the recipient, together with their metabolic and clinical condition.^2^ In lung transplant recipients, the risk of infection is higher because of direct contact with the external environment and constant exposure to pathogens.^3^

Pneumonia after lung transplantation (LTx) is a common complication. The denervation resulting from the procedure, causing impaired mucociliary clearance, added to the risk of cross-infection derived from the donor and the recipient’s airway, make pneumonia one of the leading causes of early mortality in lung transplant recipients, together with acute cellular rejection.^3^ Early pneumonia after LTx can be donor-derived pneumonia, when microorganisms are transmitted directly from the graft; recipient colonization-related pneumonia, when microorganisms previously present in the recipient infect the graft; and hospital-acquired pneumonia, as a result of long hospital stays and mechanical ventilation.^4^

Pneumonia after LTx is associated with an increased risk of 28-day and one-year mortality,^5^ ^,^ ^6^ as well as with an increased risk of antibody-mediated rejection.^7^ In addition, the repeated cycle of infection and acute cellular rejection during the first year after LTx is strongly related to the development of chronic lung allograft dysfunction, which is the main cause of long-term lung failure in such patients.^8^ ^,^ ^9^

The objective of the present study was to demonstrate the burden of pneumonia after LTx in a large center in Brazil, characterizing the main source of infection and its associated mortality. 

## METHODS

This was a retrospective cohort study of all LTx procedures performed between January of 2017 and December of 2023 at the largest public hospital in Brazil. We analyzed the general characteristics of donors and recipients. During the procedures, BAL fluid was collected from donors, and bronchial secretions were collected from donors and lung transplant recipients. In addition, BAL fluid was routinely collected from all recipients until post-LTx day 14 and was screened for pneumonia. In Brazil, only organs from brain-dead donors can be used for transplantation. 

BAL was performed in accordance with International Society for Heart and Lung Transplantation guidelines^10^ via oral bronchoscopy to avoid nasopharyngeal colonization. Two aliquots of 40 mL of sterile saline solution were instilled with the scope wedged into each lung’s middle lobe and lingula or into the lung segment with purulent secretion. Aspiration was performed immediately after instillation by vacuum suction. Bacterial, fungal, and mycobacterial cultures were performed by sowing in a specific culture medium. Microorganisms were identified by VITEK^®^ 2 (bioMérieux; Marcy-l’Étoile, France), matrix-assisted laser desorption ionization-time of flight mass spectrometry, or BD BACTEC™ MGIT™ 960 (Becton, Dickinson and Company, Franklin Lakes, NJ, USA). Antimicrobial sensitivity tests were conducted with VITEK^®^ 2, in accordance with the Clinical and Laboratory Standards Institute protocol. 

Immunosuppression induction was with 20 mg of basiliximab on postoperative days 0 and 4, as well as 500 mg of methylprednisolone, whereas the maintenance immunosuppression protocol was triple therapy with cyclosporine or tacrolimus (for lung transplant recipients < 15 years of age) plus prednisone and mycophenolate. Antimicrobial prophylaxis was with cefepime for all patients with nonsuppurative lung disease; for those with suppurative lung disease, antimicrobial prophylaxis was based on previous colonization. Patients with *Burkholderia cenocepacia* before LTx received meropenem and trimethoprim-sulfamethoxazole for 14 days or on the basis of susceptibility test results. Other antimicrobial prophylaxis at our center includes the following: universal use of 10 mg of inhaled amphotericin B twice daily; 200 mg of oral itraconazole twice daily, 5 mg/kg of ganciclovir for cytomegalovirus prophylaxis for 3 months; and trimethoprim-sulfamethoxazole for *Pneumocystis jirovecii* for lifelong prophylaxis. 

A predefined hierarchical attribution strategy based on phenotype characteristics (microbiological concordance, temporal correlation, and clinical plausibility) was applied. All cultures were systematically evaluated by two physicians separately. In case of disagreement, a third physician decided on the allocation. In situations in which the same microorganism was identified in the donor culture and in the bronchial secretions from the recipient at the time of LTx and subsequently isolated from the recipient BAL fluid within 14 days, cases were classified as mixed infections. This category was created to avoid misclassification when phenotypic data alone could not reliably distinguish between donor-derived and recipient-related transmission. 

Pneumonia was defined in accordance with the International Society for Heart and Lung Transplantation^4^ and required the presence of consistent clinical signs or symptoms; new or progressive radiological infiltrates; and microbiological confirmation. Donor-derived pneumonia was considered when the same microorganism previously found in the donor culture was also positive in the recipient BAL fluid within 14 days after LTx. Recipient colonization-related pneumonia was considered when the microorganism found in the bronchial secretions from the recipient at the time of LTx was also positive in the recipient BAL fluid within 14 days of LTx. Hospital-acquired pneumonia was defined as pneumonia occurring within 14 days after LTx and caused by a microorganism not previously identified in either the donor cultures or in the bronchial secretions from the recipient at the time of LTx, developing ≥ 48 h after hospitalization. Normal lung microbiota, such as the *Streptococcus viridans* group, *Neisseria* spp., *Corynebacterium* spp., coagulase-negative *Staphylococcus* spp., *Rothia* spp., *Enterococcus* spp., *Eikenella* spp., and *Candida* spp., was excluded from the analysis. Microorganisms with clear pathogenic relevance, particularly vancomycin-resistant enterococci (VRE), were included in descriptive microbiology and outcome analyses.^11^

Bacterial resistance patterns were defined as multidrug-resistant (MDR) if not susceptible to at least one agent in three or more antimicrobial categories; extensively drug-resistant (XDR) if susceptible to only one or two categories; and pandrug-resistant if not susceptible to any agent in any antimicrobial category.^12^ Cases of MDR bacteria that were not susceptible to carbapenem were defined as carbapenem-resistant. 

To estimate the risk of pneumonia, denominators were defined on the basis of the population at risk. Donor-derived pneumonia was calculated by using the number of recipients who received lungs from donors with positive respiratory cultures. Recipient colonization-related pneumonia was calculated by using the number of recipients colonized at the time of LTx. Hospital-acquired pneumonia was calculated by using the total number of lung transplant recipients, given that all patients were at risk of nosocomial infection after LTx. Patients were followed from LTx until death or the last available clinical follow-up. 

All statistical analyses were performed with the IBM SPSS Statistics software package, version 29.0 (IBM Corp., Armonk, NY, USA). Continuous variables were summarized as means and standard deviations or medians and interquartile ranges, depending on the data distribution. Categorical variables were expressed as absolute numbers and proportions. Survival analysis was performed by using the Kaplan-Meier method to estimate overall survival by type of pneumonia, causative microorganisms, and antimicrobial resistance patterns. Differences between survival curves were assessed with the log-rank test. Comparisons between categorical variables were performed with the chi-square test or Fisher’s exact test, as appropriate. A two-sided value of p < 0.05 was considered significant. 

The study was approved by the Human Research Ethics Committee of the *Instituto do Coração do Hospital das Clínicas da Universidade de São Paulo* (Protocol no. 64097922.2.0000.0068), located in the city of São Paulo, Brazil. 

## RESULTS

During the study period, 217 LTx procedures were performed. Of those, 159 (73.2%) were bilateral. Only 33 donors (15.2%) had pneumonia reported on the donor form before or during organ procurement; however, 61.8% were receiving antibiotics. The mean donor intubation time was 3.7 ± 2.1 days. Regarding the recipients, 58 (26%) were hospitalized before the procedure, and 22 (10.1%) were in the ICU. The general characteristics of donors and recipients are presented in Table 1, whereas the characteristics associated with the different types of pneumonia are shown in Table 2. 

**Table 1:** Characteristics of donors and recipients.^a^

DONORS	n = 217	RECIPIENTS	n = 217
Male	136 (62.7)	Male	93 (42.9)
Age, years	30.2 ± 12.1	Age, years	40.6 ± 16.3
Median intubation time, days	3.7 (2.2)	Type of transplantation	
PaO_2_/FiO_2_ at the time of offer	387.9 ± 81.8	Unilateral	58 (26.7)
Cause of death		Bilateral	159 (73.3)
Trauma	126 (58.1)	Pretransplant status	
Stroke	74 (34.1)	Not hospitalized	159 (73.3)
Anoxia	11 (5.1)	Not in the ICU	36 (16.6)
Tumor	4 (1.8)	Admitted to the ICU	22 (10.1)
Other	2 (0.9)	Disease	
Comorbidities		Suppurative	89 (41)
Smoking	21 (9.7)	Fibrosis	67 (30.9)
Cocaine	11 (5.1)	Obstructive	29 (13.4)
Alcohol	13 (6)	Circulation	9 (4.1)
Diabetes	7 (3.2)	Connective tissue disease	7 (3.2)
Pre-harvest status		Other	16 (7.4)
Use of vasopressors	205 (94.5)	Recipient BAL fluid with positive culture	107 (49.3)
Pneumonia	33 (15.2)	BAL fluid positive for bacterial culture	145 (100)
Use of antibiotics	133 (61.8)	*Pseudomonas aeruginosa*	68 (47.2)
Secretion at bronchoscopy	46 (21.3)	*Staphylococcus aureus*	33 (22.9)
Donor BAL fluid with positive culture	149 (68.6)	*Stenotrophomonas maltophilia*	12 (8.3)
BAL fluid positive for bacterial culture	318 (100)	*Burkholderia cepacia*	8 (5.6)
*Staphylococcus aureus*	116 (36.5)	*Acinetobacter baumannii*	4 (2.8)
*Pseudomonas aeruginosa*	33 (10.4)	Other	18 (12.9)
*Klebsiella pneumoniae*	27 (8.5)		
*Acinetobacter baumannii*	25 (7.9)		
*Haemophilus influenzae*	21 (6.6)		
*Enterobacter cloacae*	16 (5.0)		
Other	79 (25.1)		

a Data presented as n (%) or mean ± SD.

**Table 2:** Characteristics of donors and recipients, by type of pneumonia.

	Negative n (%)	Recipient colonization-related pneumonia* n (%)	Donor-derived pneumonia* n (%)	Mixed** n (%)	Hospital-acquired pneumonia* n (%)	p
Total	173 (80.8)	14 (13.1%)	8 (5.36%)	2 (0.9%)	17 (7.83%)	
DONOR CHARACTERISTICS						
Male	103 (75.7)	11 (8.1)	6 (4.4)	2 (1.5)	14 (10.3)	0.149
Cause of brain death						0.943
Trauma	98 (45.8)	10 (4.7)	7 (3.3)	2 (0.9)	8 (3.7)	
Stroke	59 (27.6)	4 (1.9)	1 (0.5)	0 (0)	8 (3.7)	
Anoxia	10 (4.7)	0	0	0	1 (0.5)	
Tumor	2 (0.9)	0	0	0	0	
Other	4 (1.9)	0	0	0	0	
Tobacco use	18 (9.0)	1 (0.5)	2 (1.0)	0	0	0.385
Cocaine use	9 (4.4)	0	1 (0.5)	0	1 (0.5)	0.799
Alcohol use	10 (5.0)	0	0	1 (0.5)	2 (1.0)	0.74
Intubation for more than 3 days	76 (35.8)	7 (3.3)	6 (2.8)	1 (0.5)	5 (2.4)	0.313
PaO_2_/FiO_2_ at the time of offer < 400	103 (48.1)	2 (0.9)	2 (0.9)	1 (0.5)	6 (2.8)	0.003
Pneumonia at the time of harvest	29 (13.7)	1 (0.5)	0	0	3 (1.4)	0.573
Antibiotic use at the time of harvest	109 (51.7)	8 (3.8)	6 (2.8)	0	10 (4.7)	<0.001
Secretions at bronchoscopy	37 (19.4)	1 (5.2)	0	1 (0.5)	7 (3.7)	0.083
RECIPIENT CHARACTERISTICS						
Male	66 (30.8)	9 (4.2)	3 (1.4)	1 (0.5)	11 (5.1)	0.107
Type of transplant						0.158
Unilateral	47 (22.0)	6 (2.8)	3 (1.4)	1 (0.5)	1 (0.5)	
Bilateral	126 (58.9)	8 (3.7)	5 (2.3)	1 (0.5)	16 (7.5)	
Pretransplant status						0.935
Not hospitalized	126 (58.9)	10 (4.7)	6 (2.8)	2 (0.9)	14 (6.5)	
Not in the ICU	28 (13.1)	2 (0.9)	2 (0.9)	0	2 (0.9)	
Admitted to the ICU	19 (8.9)	2 (0.9)	0	0	1 (0.5)	
Pretransplant disease						0.323
Suppurative	64 (29.9)	12 (5.6)	4 (1.9)	1 (0.5)	7 (3.3)	
Fibrosis	54 (25.2)	1 (0.5)	2 (0.9)	0	8 (3.7)	
Obstructive	24 (11.2)	1 (0.5)	2 (0.9)	1 (0.5)	1 (0.5)	
Pulmonary Hypertension	9 (4.2)	0	0	0	0	
Other	22 (10.3)	0	0	0	1 (0.5)	
Postoperative PGD						0.027
Grade 0-2	126 (60.6)	12 (5.8)	7 (3.4)	0	12 (5.8)	
Grade 3	43 (20.7)	0	1 (0.5)	2 (1.0)	5 (2.4)	
Postoperative ECMO	24 (11.2)	2 (0.9)	3 (1.4)	0	4 (1.9)	0.337

PGD: primary graft dysfunction; and ECMO: extracorporeal membrane oxygenation. *The proportions were calculated by using population-at-risk denominators, as follows: donor-derived pneumonia, n = 149; recipient colonization-related pneumonia, n = 107; and hospital-acquired pneumonia, n = 217. **Considered when pathogens from the donor and the recipient were present on postoperative day 14.

From a total of 217 cultures, 318 different bacteria and 12 fungi were found in 149 donors. The most prevalent bacterial agents were *S*. *aureus* (36.5%) and *Pseudomonas aeruginosa* (10.4%). Of the total of bacteria, 59 (18.5%) showed patterns of resistance: 6.3% were methicillin-resistant *S*. *aureus*; 6.6% were MDR gram-negative rods; 8.1% were carbapenem-resistant bacteria; and 0.7% were VRE. Fungal culture showed *Aspergillus spp*. (58.3%), zygomycetes (16.7%), *Cryptococcus neoformans* (8.3%), and *Trichosporon asahii* (8.3%)*.*

**Figure 1:**
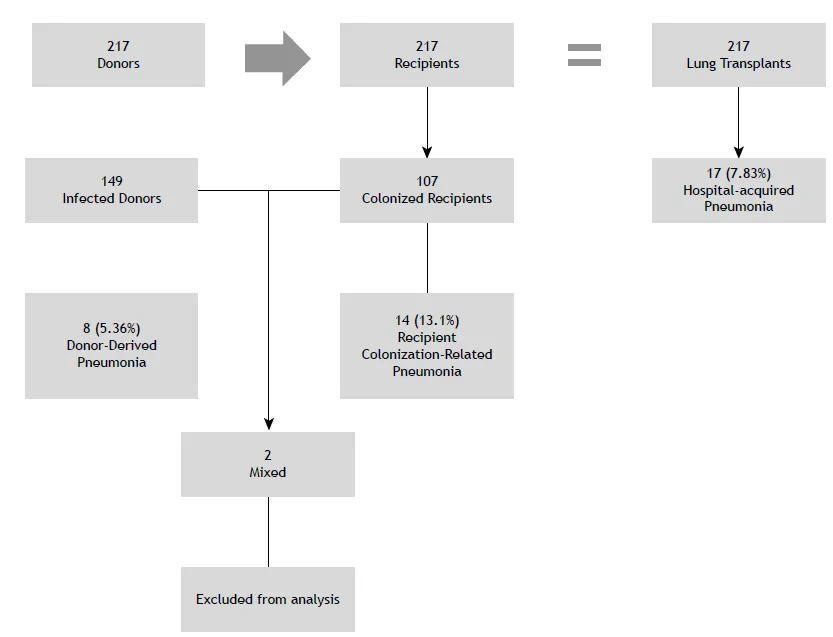
Flow diagram illustrating the study population and classification of early pneumonia (≤ 14 days after lung transplantation) in accordance with the presumed source. Cases with overlapping donor and recipient microbiology (“mixed”) were excluded from the source attribution analysis.

A total of 107 recipients had been colonized before LTx. The most prevalent bacteria were *P*. *aeruginosa* (in 46%), followed by *S*. *aureus* (in 22%) and *Stenotrophomonas maltophilia* (in 7.5%). The main fungi found were *Aspergillus* spp. (in 43%). One patient presented with a culture of *Fusarium spp*. Four mycobacterial cultures were positive: two with *M*. *intracellulare*, one with *M*. *fortuitum*, and one with *M. avium*. The characteristics of the study population and the classification of pneumonia cases are summarized in Figure 1. 

### Donor-derived pneumonia

Within 14 days after LTx, 39 patients had positive BAL cultures, yielding a total of 50 bacterial isolates. Donor-derived pneumonia was identified in 8 of 149 recipients who received lungs from donors with positive respiratory cultures (5.36%), all due to gram-negative rods: four *Klebsiella pneumoniae* (50%), three *P*. *aeruginosa* (37.5%), and one *Acinetobacter baumannii* (12.5%). Two (25%) were MDR; four (50%) were carbapenem-resistant; and one was XDR. 

### Recipient colonization-related pneumonia

Recipient colonization-related pneumonia occurred in 14 of 107 previously colonized recipients (13.1%), involving 18 different bacterial isolates. Three patients underwent unilateral LTx and had identification of colonizing agents only by intraoperative bronchial secretion cultures. Four patients were hospitalized prior to surgery, and two were in the ICU. The most prevalent microorganisms were *P*. *aeruginosa* (in 58.8%) and *S*. *aureus* (in 11.8%). Fourteen microorganisms presented patterns of resistance: 27.7% were carbapenem-resistant; 22.2% were MDR; and all *S*. *aureus* were methicillin-resistant. 

### Hospital-acquired pneumonia

Hospital-acquired pneumonia was identified in 17 of 217 lung transplant recipients (7.83%), being caused by 20 different bacterial isolates; only three patients were hospitalized before LTx, and only one was in the ICU. On postoperative day 14, 8 patients (47%) were still in the ICU. The most prevalent bacteria were *A*. *baumannii* (in 20%), followed by *P*. *aeruginosa* (in 15%), *K*. *pneumoniae* (in 10%), *Stenotrophomonas maltophilia* (in 10%), and *B*. *cepacia* complex (in 10%). Twelve agents showed patterns of resistance: 41.6% were MDR; 41.6% were carbapenem-resistant; and 8.3% were XDR. 

### Mortality

**Figure 2:**
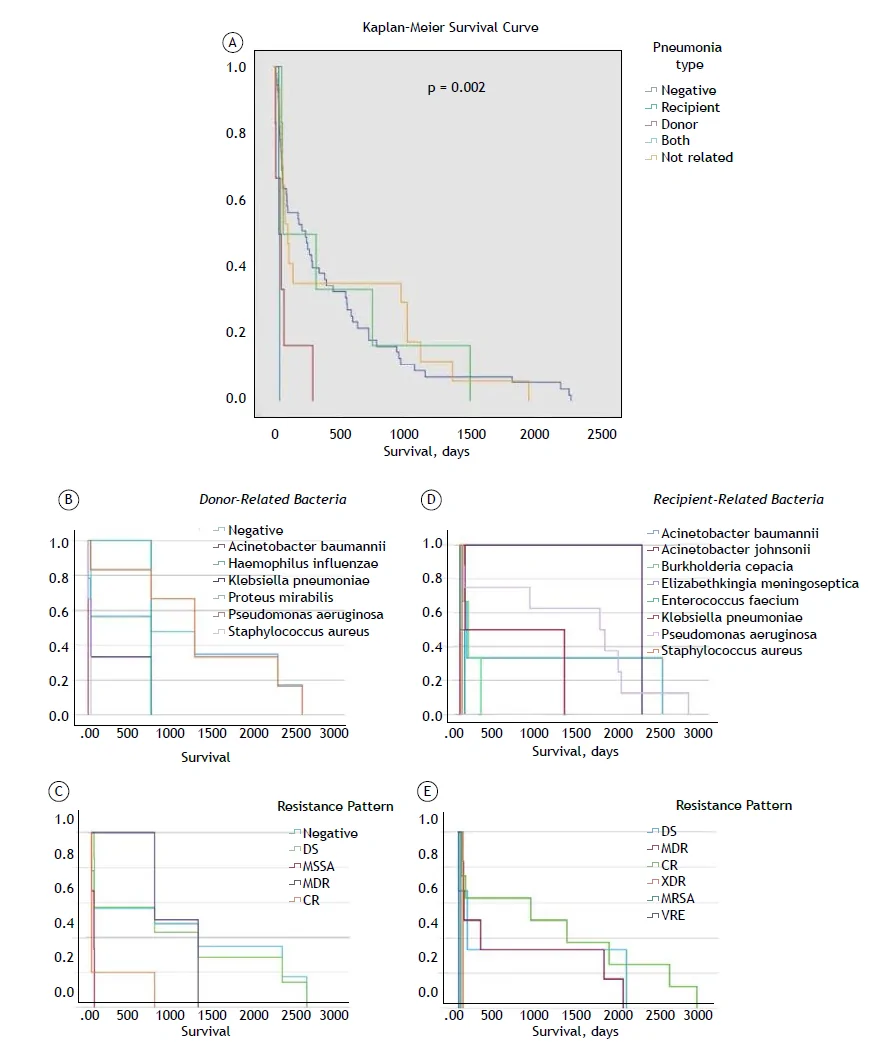
Kaplan-Meier survival curves, by pneumonia source and microbiological characteristics. In A, overall survival, by type of pneumonia. In B and C, survival by donor microbiology and resistance patterns. In D and E, survival by recipient microbiology and resistance patterns. Censoring marks are displayed on all curves. Differences in follow-up duration between subgroups lead to curve termination at different time points; therefore, apparent survival drops at late time points reflect censoring rather than complete mortality. DS: drug-sensitive; MDR: multidrug-resistant; CR: carbapenem-resistant; XDR: extensively drug-resistant; MSSA: methicillin-sensitive *Staphylococcus aureus*; MRSA: methicillin-resistant *Staphylococcus aureus*; and VRE: vancomycin-resistant enterococci.

The mean follow-up period was 810 days. Death related to donor-derived pneumonia occurred in 4 of 8 patients (50%). Fatal cases involved infections caused by *K*. *pneumoniae* and carbapenem-resistant gram-negative microorganisms. Regarding recipient colonization-related pneumonia, 4 patients died (28.6%), with a higher tendency in those infected with *P*. *aeruginosa.* Mortality associated with hospital-acquired pneumonia was 50%, being higher in those infected with *S*. *aureus*, *A*. *johnsonii*, and *E*. *faecium* (p < 0.001); most of the cases were associated with methicillin-resistant *S*. *aureus*, VRE, or XDR bacteria (p = 0.008). In the overall survival analysis, Kaplan-Meier curves demonstrated a significant difference in survival across pneumonia source categories (log-rank test, p = 0.002; Figure 2), with lower survival among recipients with donor-derived pneumonia. 

## DISCUSSION

In our study, we found that although infrequent, donor-derived pneumonia was associated with high early mortality, comparable to that observed in hospital-acquired pneumonia. Donor-derived infection is a risk to all solid organ transplant recipients, particularly lung transplant recipients, given that all brain-dead donors are on mechanical ventilation and hospitalized for several days in the ICU prior to organ procurement. 

The rate of donor-derived pneumonia varies widely across regions and countries. A recent cohort study^13^ including 183 patients from Saudi Arabia found a 23.9% incidence of donor-derived pneumonia, most of the patients presenting with gram-negative bacilli and MDR pathogens. In a study conducted in Italy^14^ and including 169 lung transplant recipients, there were 14 cases of donor-derived pneumonia, despite a predominance of gram-positive pathogens. Our cohort showed a lower incidence of donor-derived pneumonia (5.36% of those who received a lung from an infected donor), with a predominance of gram-negative bacteria and carbapenem-resistant pathogens, possibly due to the efficacy of our antimicrobial prophylaxis. 

Despite a low incidence of donor-derived pneumonia, our analysis found a higher risk of death in recipients with donor-derived pneumonia, showing a trend toward worse outcomes in those patients, similar to those reported elsewhere, including higher odds of primary graft dysfunction^14^; inferior early graft function, especially in previously uncolonized recipients^13^; and significantly longer duration of mechanical ventilation and ICU length of stay.^14^ ^,^ ^15^

In our cohort, donor carbapenem-resistant pathogens were more frequently associated with recipient infection than were susceptible organisms. This finding may be influenced by the institutional antimicrobial prophylaxis protocol with cefepime, which effectively covers most of the drug-susceptible and short-resistant gram-negative and gram-positive bacteria, potentially reducing transmission. Prior studies have suggested that the presence of MDR bacteria in donor cultures is not necessarily associated with a higher risk of donor-recipient transmission when appropriate antimicrobial strategies are applied.^16^ ^-^ ^18^ However, once donor-derived transmission occurs, infections caused by carbapenem-resistant pathogens have been associated with worse early outcomes, including increased mortality.^13^

Nevertheless, given the shortage of donor lungs, should such outcomes be used in order to determine organ usability? Evidence of donor lung infection was once considered an absolute contraindication to LTx.^19^ Recent studies have shown that the use of such lungs is possible in the setting of appropriate prophylaxis and treatment, if required.^13^ ^,^ ^18^ Additionally, donor BAL culture is not always available prior to procurement to support this decision, and the presence of lung infection may not correlate with radiology or bronchoscopy findings. Our study supports that donor lungs that are known to be infected with MDR or XDR bacteria should be considered unsuitable for LTx, especially for uncolonized recipients.^14^ Nevertheless, mild pneumonia caused by bacteria with no resistance pattern can be considered for LTx if all other criteria of acceptability are met. The evaluation of a donor lung is complex and should be based on multiple factors concerning donor and recipient characteristics.^20^ ^,^ ^21^

Interestingly, in our analysis, recipient colonization-related pneumonia was not associated with unilateral LTx or previous hospitalization, as would be expected. In a study conducted in Italy,^22^ recipient-related colonization was shown to be an independent predictor of MDR bacterial infection after LTx (hazard ratio = 2.48; 95% CI, 1.04-5.90; p = 0.04). Analyses based on patient outcome are inconclusive. Although some studies have shown no correlation with early graft dysfunction,^14^ others have found a higher one-year mortality rate.^23^ In our analysis, most transmissions happened within *P*. *aeruginosa* colonized patients, who also showed increased mortality. 

Hospital-acquired pneumonia is an undesirable complication related to the long-term mechanical ventilation and hospital stay that some lung transplant recipients require. In our study, only 7.83% of lung transplant recipients presented with hospital-acquired pneumonia, and 47% of those were still in the ICU on postoperative day 14. A similar proportion was found in a recent study conducted in Canada, where 9.3% of lung transplant recipients presented with ventilator-associated pneumonia or hospital-acquired pneumonia within 30 days after LTx.^24^ However, unlike our study, the study conducted in Canada showed no association between hospital-acquired pneumonia and higher mortality.^24^ In a large study of a Spanish database^25^ including 4,598 lung transplants, a significant increase in mortality was found in patients with hospital-acquired pneumonia when compared with those without (29.7% vs. 13.3%; p < 0.001), with an odds ratio of 2.97 (95% CI, 2.24-3.94). 

The present study has inherent limitations that are due to its retrospective design and single-center nature. Pathogen attribution was based on phenotypic identification and antimicrobial susceptibility patterns, given that molecular typing was not routinely available. However, to reduce the risk of bias, all data were ostensibly reviewed by more than one researcher at different times. All pathogens were verified by infectious disease specialists and were considered only if virulence and quantification criteria were met. In addition, causes of death were not systematically evaluated; therefore, mortality outcomes reflected overall mortality. 

Pneumonia remains an important cause of death in the first month after LTx. Although infrequent, donor-derived pneumonia, especially donor-derived pneumonia caused by MDR pathogens, is associated with a higher risk of mortality and should be taken into consideration when evaluating a donor lung. In addition, recipient colonization-related and hospital-acquired pneumonia are linked to an increased mortality risk, especially in patients colonized with *P*. *aeruginosa* and those with XDR bacteria, respectively. 
